# Differentially expressed transcripts associated with depressive symptoms during pregnancy and postpartum

**DOI:** 10.1038/s41380-025-03068-z

**Published:** 2025-06-05

**Authors:** Richelle D. Björvang, Maria Vrettou, Xabier Bujanda Cundin, Eugenio Del Prete, Joëlle Rüegg, Susanne Lager, Diego di Bernardo, Erika Comasco, Alkistis Skalkidou

**Affiliations:** 1 Department of Women’s and Children’s Health, Uppsala University, 751 85 Uppsala, Sweden; 2 Department of Clinical Science, Intervention and Technology, Karolinska Institutet, Karolinska University Hospital Huddinge K65, SE-14157 Huddinge, Sweden; 3 Department of Women’s and Children’s Health, Science for Life Laboratory, Uppsala University, BMC, POB 593, 75124 Uppsala, Sweden; 4 Telethon Institute of Genetics and Medicine, Via Campi Flegrei 34, 80078 Pozzuoli, Italy; 5 Department of Organismal Biology, Uppsala University, 752 36 Uppsala, Sweden; 6 Department of Chemical, Materials and Industrial Engineering, University of Naples “Federico II”, Naples, Italy

**Keywords:** Depression, Molecular biology

## Abstract

Peripartum depression can have severe impact on the mother’s and the infant’s health. Yet, its biological underpinnings are largely unknown. The present study sought to identify transcriptomic signatures of depressive symptoms during pregnancy and postpartum. Blood samples were collected during late pregnancy or early postpartum for mRNA isolation and sequencing, while depressive symptoms were assessed using the Edinburgh Postnatal Depression Scale (EPDS). Based on the timepoint when the samples were collected, differentially expressed genes (DEGs) were identified by (1) comparing mRNA levels between the depression symptom trajectory groups, and (2) correlating with EPDS scores. DEGs for samples collected during late pregnancy, but not postpartum, were associated with depressive symptoms occurring only during pregnancy or persisting postpartum, compared with controls. There were 16 upregulated and 109 downregulated DEGs significantly associated with EPDS score at week 32 among samples collected during late pregnancy. Gene Set Enrichment Analysis identified immune response and cell motility as processes linked to these DEGs. Hypothesis-based analysis on previously identified postpartum depressive symptoms-related DEGs replicated a positive association between expression of immune-related genes *ISG15* and *RSAD2* with postpartum-onset depressive symptoms, both in samples taken during late pregnancy and postpartum. The present findings point to transcriptomic signatures associated with peripartum depressive symptoms, mostly related to immune system dysregulation.

## Introduction

Peripartum depression during pregnancy or postpartum affects about 12–17% of all females giving birth [1–4], leading not only to great suffering for the female [5], but also having negative implications for the development and mental health of the child [6]. Notably, suicide is currently the leading cause of maternal mortality in high-income countries [7].

The attempts to identify biomarkers for psychiatric phenotypes have increased during the last decade, especially supported by the use of high-throughput omics techniques [8]. Besides self-reported psychosocial characteristics, biophysiological markers have been associated with peripartum depression as well. Such markers include estrogen receptor signalling, immune system parameters, stress hormones and reactivity, as well as stress-related genetic polymorphisms [9–17]. On the transcriptome level, differentially expressed genes (DEGs) during pregnancy, among which genes implicated in estrogen signaling [18] and the stress response system [19], were associated with peripartum depression, and later validated [20]. Recently, lymphoblastoid cell lines, derived from females with and without past postpartum depression (PPD), were experimentally exposed to hormonal conditions mimicking the peripartum milieu [21]. Innate downregulation of genes (i.e. mRNA expression) involved in cellular stress signaling and homeostasis maintenance was observed especially in those cell lines from women with past PPD, and treated with estrogen and progesterone add-back [21]. Moreover, transcriptional differences during the third pregnancy trimester [20] and at week six postpartum [9, 22] linked to altered immune response have been identified in subjects with depressive symptoms postpartum. Lastly, extracellular vesicle mRNA levels throughout pregnancy and postpartum have been found altered among those who went on to develop PPD, and this alteration, mainly seen in mid and late pregnancy, was associated with decreased autophagy [23].

Altogether, as summarized in Table S1, these findings point to an association between peripartum depression and dysregulation of the expression of several genes during the peripartum period, predominantly implicated in stress, gonadal steroid hormones and immune-related pathways. Nonetheless, as previous transcriptomic studies employed case-control designs with limited sample sizes, assessed transcripts and depression status concurrently rather than longitudinally, and/or predominantly included individuals with a history of mood disorders, it is especially challenging to derive definitive conclusions regarding gene expression patterns and regulatory mechanisms. Furthermore, distinct trajectories based on onset and persistence of symptoms within the peripartum period (i.e., antepartum depression, postpartum-onset depression, and persistent depression) point to different risk factors [24] but knowledge is lacking about biological factors influencing individual susceptibility to these different trajectories of peripartum depression.

**Fig. 1:**
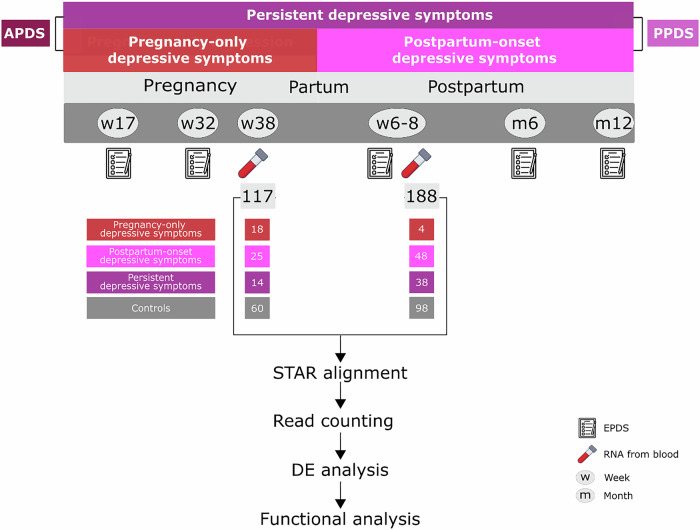
Schematic presentation of the study design. The data was used to compared the four distinct trajectory groups (controls vs. pregnancy-only depression vs. postpartum-onset depression vs. persistent depression); to compare women with depression during pregnancy (pregnancy-only and persistent group) vs controls and then women depressed postpartum (postpartum-onset and persistent group) vs controls; as well as to assess the EPDS score as a continuous variable. APD Depression during pregnancy which could be persistent or resolved postpartum, PPD depression postpartum which could be persistent or postpartum-onset depression, STAR Spliced Transcripts Alignment to a Reference, DE Differential Expression.

The present study aimed to identify transcriptomic markers of peripartum depression (Fig. 1). Specifically, DEGs were investigated among the trajectories of peripartum depression, with controls as the reference. Moreover, correlations between the severity of peripartum depression symptoms and gene expression were investigated to identify quantitative relationships. As secondary aims, transcriptome differences were investigated in relation to depression as a binary outcome at each timepoint, to facilitate comparison of results with previous studies. Transcripts previously associated with PPD, considering Edinburgh Postnatal Depression Scale (EPDS) scores, were investigated as hypothesis-based analyses [20]. Lastly, we explored differences in genes expressed in the brain during the peripartum period [25, 26].

## Methods

### Study population and biological sampling

This study stemmed from the Biology, Affect, Stress, Imaging and Cognition (BASIC) study, a large longitudinal population-based prospective cohort, conducted from 2009–2019 in Uppsala, Sweden [27]. This cohort comprised of 6478 pregnancies in 5492 women. Briefly, women undergoing routine ultrasound at gestational weeks 16–18 at the Uppsala University Hospital were invited to participate in the study. Exclusion criteria in the BASIC cohort were: (i) age under 18 years, (ii) non-viable pregnancy at ultrasound, (iii) blood borne infections, (iv) difficulties to read and understand Swedish, and (v) protected identity (i.e. have personal data withheld from public registers and have restricted access). For the study in this paper, additional exclusion criteria were: (a) did not provide RNA samples, (b) pregnant with twins and (c) smoking during sample collection. The women were followed at gestational week 32 as well as 6 weeks, 6 months and 12 months postpartum. At each timepoint, the women were asked to fill out web-based questionnaires, which included sociodemographic information (e.g. age, marital status, education, employment), gynecologic and obstetric information (e.g. parity, mode of delivery, premature birth), lifestyle, sleep, psychiatric-related (e.g. history of depression or previous contact with a psychologist, anxiety during pregnancy and postpartum), trauma and stressful events, among others. They were also asked about current medications, where they entered their answers as free-text. Information on use of selective serotonin reuptake inhibitors (SSRI) was taken from this question. Information on diagnoses and other relevant variables from national registers were used for information on socioeconomic and medical data, respecitively. Subgroups of participants were invited for an additional visit in gestational week 38 and/or postpartum week 8 where blood was collected. To oversample women with ongoing depressive symptoms, women with EPDS scores 12 or more [12] in gestational week 32 or postpartum week 6 and/or reporting antidepressant treatment were prioritized. Among the 715 and 713 invitations sent out for pregnancy and postpartum additional visits, 349 and 413 consented to participate in the pregnancy and postpartum visits, respectively. In this nested case-control group, peripheral blood samples were collected into PAXgene tubes and stored at −80 °C until RNA extraction. As the planned analyses were exploratory, sample size was set in line with other studies in the field, as no formal sample size calculation could be performed. Among those with complete data and RNA samples provided, 117 women with RNA samples taken during pregnancy and 188 women with RNA samples taken during postpartum were included in this study, as illustrated in the flowchart in Figure S1. Informed consent was obtained from all participants. This study was approved by the Uppsala Regional Ethical Committee (Dnr 2009/171) and carried out according to the Principles of the Declaration of Helsinki [28].

### Symptoms of peripartum depression

Depressive symptoms during pregnancy (week 17, week 32, and week 38) and postpartum (week 6, week 8 and month 6) were assessed through EPDS [12], a screening tool for depressive symptoms during the peripartum period [29]. The cut-off value of EPDS used was 13 and higher during pregnancy [30], and 12 and higher during postpartum [29], in line with the two respective validation studies in Sweden. Furthermore, continuous scores of EPDS at week 32 and postpartum week 6 were also considered.

### RNA sequencing

As described in detail in the supplement, RNA was extracted using the PAXgene 96 Blood RNA Kit (Qiagen GmbH) and prepared for sequencing using QuantSeq 3′ mRNA-Seq Library Prep Kit FWD (Lexogen). The RNA libraries were sequenced as 1 × 100 bp reads on Illumina NovaSeq 6000 S2 Flow CellSystem and aligned to the UCSC Genome Browser hg38 using *Spliced Transcripts Alignment to a Reference* (STAR) with standard parameters. Finally, the count matrix per gene was obtained for each sample using *htseq-count* function from *HTSeq* [31]. Data are available on Gene Expression Omnibus database repository with the GEO Accession code GSE290313.

### Statistical analyses

Analyses were stratified according to the timepoint the sample was taken, i.e. pregnancy and postpartum. For the samples provided postpartum, the trajectory group with depressive symptoms only during pregnancy was excluded as it was small (*n* = 4). Furthermore, controls taking SSRI during blood collection (*n* = 2) were excluded from further analyses to ensure homogeneity of the healthy control group, and avoid depressed individuals near remission. Secondary analyses were also performed after excluding SSRI users during blood collection in the depressed groups (*N* = 5 for samples collected during pregnancy, and *N* = 13 for samples collected during postpartum). Only protein-coding genes were included in the analyses. The minimum raw count of reads was set to 50 in at least 12 samples. Data quality was assessed with the analyses of raw counts distribution per sample, and of possible outlier genes per sample by considering Cook’s distance. Age and pre-pregnancy BMI were used as covariates as they are expected to impact both the transcriptome and outcome [32–36]. The outcomes of interests were: (1) trajectories of peripartum depressive symptoms with controls as the reference, (2) EPDS score at pregnancy week 32 (for pregnancy samples only), (3) EPDS score at postpartum week 6, (4) antepartum depressive symptoms (APDS) vs no APDS (for pregnancy samples only), and (5) postpartum depressive symptoms (PPDS) vs no PPDS. Differential expression analysis was based on the normalized counts, resulting in the lists of DEGs in terms of logarithm in base 2-fold change (logFC) between the compared pairwise conditions. Statistical significance was based on *p*-values calculated with the Wald’s test and corrected for the multiple comparisons with the Benjamini-Hochberg method. The negative binomial generalized linear models were adjusted for age and BMI to allow the least biased estimation of net effects of the biological factors. Gene Ontology Enrichment Analysis (GOEA) with Gene Ontologies Biological Processes (GO_BP) and Molecular Functions (GO_MF) were performed on significant DEGs (adjusted *p*-value < 0.05) using *enrichGO* function in the R package *clusterProfiler*. Similarly, Kyoto Encyclopedia of Genes and Genomes (KEGG) pathway over-representation analyses were performed using *enrichKEGG* function in the R package *clusterProfiler*. Gene Set Enrichment Analysis (GSEA) of genes ranked according to logFC were determined using the R package *gep2pep* to identify enriched GO_BP, GO_MF, and KEGG pathways.

Replication of findings on previously identified DEGs associated with PPDS was also performed. Subsets of significant postpartum depression candidate genes published by [20] were extracted (*N* = 38 genes for PPD; *N* = 71 genes for PPDS or continuous EPDS scores). Herewith, this set will be referred to as “PPD genes” and “EPDS genes”, respectively. Moreover, to investigate if there were genes expressed in blood that were also expressed in the brain tissue, we combined the 700 genes showing significant changes in gene expression across different brain regions during postpartum [25], with the 181 genes that were expressed both in the female brain and in extracellular vesicles circulating in maternal blood during the peripartum period [26]. Because there were 12 genes that were present in both sets, this gene set resulted to 869 genes and will be referred to as “CNS genes”.

Analysis of transcriptomic data was performed in R environment (ver 4.3.1) [37] through *RStudio IDE* (ver 2023.12.1 Build 402) [38] using *DESeq2* (ver 1.40.2) [39] for variance stabilizing transformation (VST), data normalization and differential expression analysis, *clusterProfiler* (ver 4.8.2) [40] for GOEA and *gep2pep* (ver 1.22.0) [41] for GSEA, *biomaRt* [42] to access ENSEMBL BioMart for functional annotation, and *msigdbr* [43] packages for the molecular signature database gene sets for GSEA.

## Results

### Sample characteristics

**Fig. 2:**
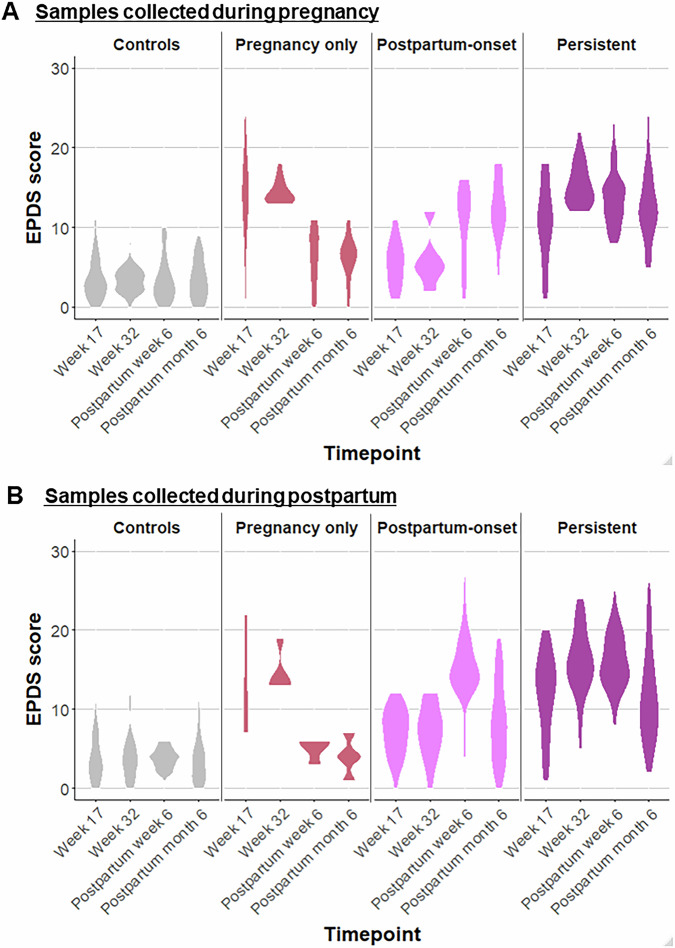
EPDS scores of the depressive symptoms trajectories. Violin plots show the distribution of the EPDS scores at gestational weeks 17 and 32 and postpartum week 6 and month 6 for samples collected during pregnancy **A** and postpartum **B**.

Out of 117 participants with samples collected during pregnancy, 18 (15.3%) had depressive symptoms only during pregnancy, 14 (12%) had postpartum-onset depressive symptoms and 25 (21.4%) had persistent depressive symptoms. Out of 188 participants with samples collected postpartum, 4 (2.1%) had depressive symptoms only during pregnancy, 48 (25.5%) had postpartum-onset depressive symptoms and 38 (20.2%) had persistent depressive symptoms. The characteristics of the subjects are presented in Tables S2 and S3, while the EPDS scores among the different trajectories in samples collected during pregnancy and postpartum are shown in Fig. 2A and B, respectively.

### DEGs in relation to trajectories of peripartum depression symptoms

DEGs were investigated among the trajectories of peripartum depression: i) controls (no depressive symptoms at any timepoint throughout pregnancy and postpartum), ii) depressive symptoms during pregnancy only (depressive symptoms during pregnancy only and resolved postpartum), iii) postpartum-onset depressive symptoms (depressive symptoms with postpartum-onset), and iv) persistent depressive symptoms (depressive symptoms in at least one timepoint during pregnancy and in at least one timepoint during postpartum).

**Fig. 3:**
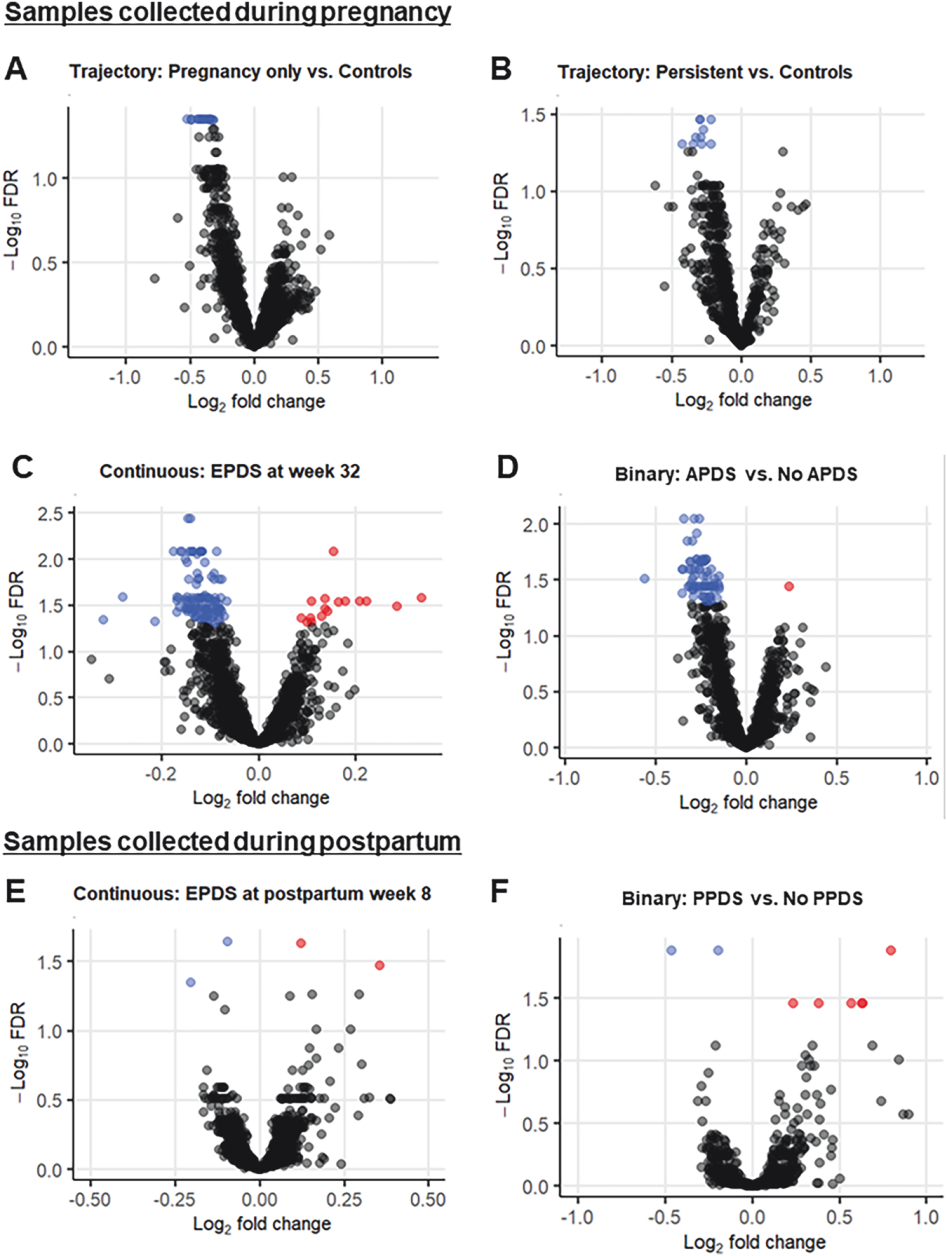
DEGs in peripartum depression outcomes. Volcano plots showing up- and downregulated genes in samples collected during pregnancy **A**–**D** and postpartum **E,** **F** when investigating several peripartum depression outcomes. Investigating peripartum depression trajectories, **A** and **B** show genes associated with depressive symptoms only during pregnancy, and persistent depressive symptoms, respectively, in samples collected during pregnancy. **C** shows upregulated and downregulated DEGs significantly associated with EPDS score at week 32. **D** shows DEGs between APDS and no APDS. In samples collected postpartum, **E** shows DEGs for EPDS score at postpartum week 8 while **F** shows DEGs between PPDS and no PPDS. There were no significant DEGs in postpartum-onset depressive symptoms vs.  controls for both timepoints. Red color indicates significant DEGs that are up- regulated (logFC > 0); blue indicates down-regulated (logFC < 0) significant DEGs, while grey color indicates non-significant genes.

**Fig. 4:**
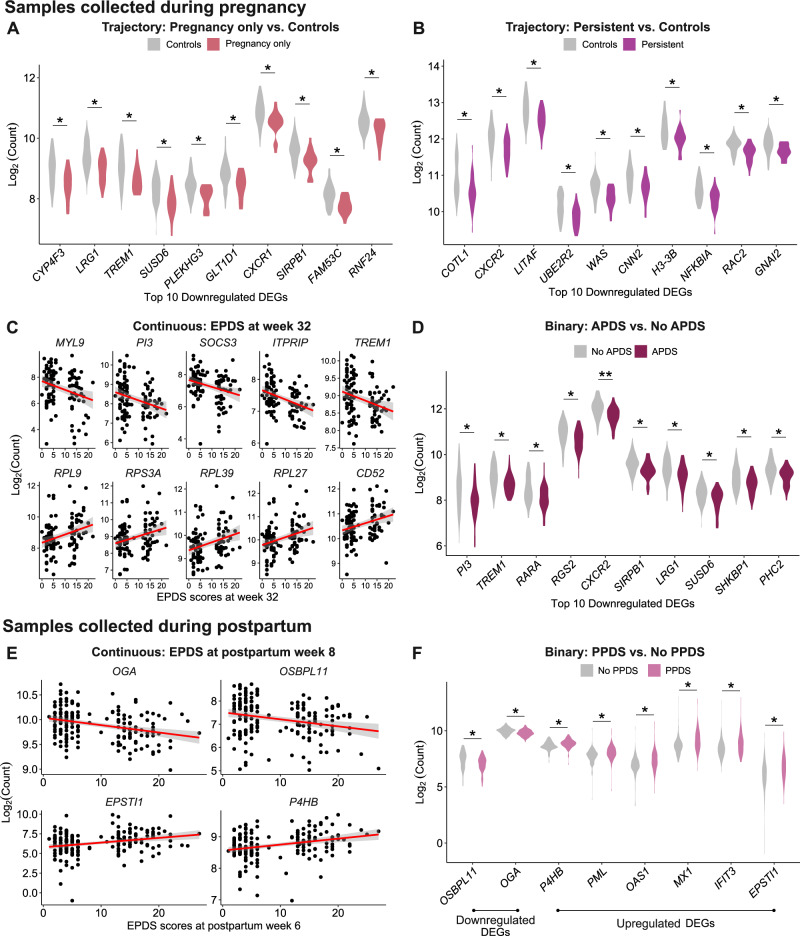
Top DEGs in peripartum depression outcomes. Violin plots (for binary outcomes) and scatter plots (for continuous outcomes) showing significant up- and downregulated genes in samples collected during pregnancy **A**–**D** and postpartum **E,** **F** when investigating several peripartum depression outcomes. Investigating peripartum depression trajectories, **A,** **B**, and **C** show top 10 DEGs (based on decreasing logFC) associated with depressive symptoms only during pregnancy, and persistent depressive symptoms, respectively, in samples collected during pregnancy. **C** shows top 5 upregulated and downregulated DEGs for EPDS score at week 32 while **D** shows top 10 DEGs between APDS and no APDS. In samples collected postpartum, **E** shows DEGs for EPDS score at postpartum week 8 while **F** shows DEGs between PPDS and no PPDS. There were no significant DEGs in postpartum-onset depressive symptoms vs. controls for both timepoints. FDR: False Discovery Rate (* FDR < 0.05, ** FDR < 0.01).

Among the individuals with samples collected during pregnancy, those with depressive symptoms only during pregnancy showed significant downregulation of 18 genes (Figs. 3A and 4A, Supplementary Excel File), while individuals with persistent depressive symptoms showed significant downregulation of 10 genes compared to controls (Figs. 3B and 4B, Supplementary Excel File). Among those, only the gene *CNN2* was downregulated both in individuals with antepartum-only and persistent depressive symptoms. There were no significant DEGs for postpartum-onset depressive symptoms compared to controls. LogFC of DEGs was in the range [−0.53, −0.21].

**Fig. 5:**
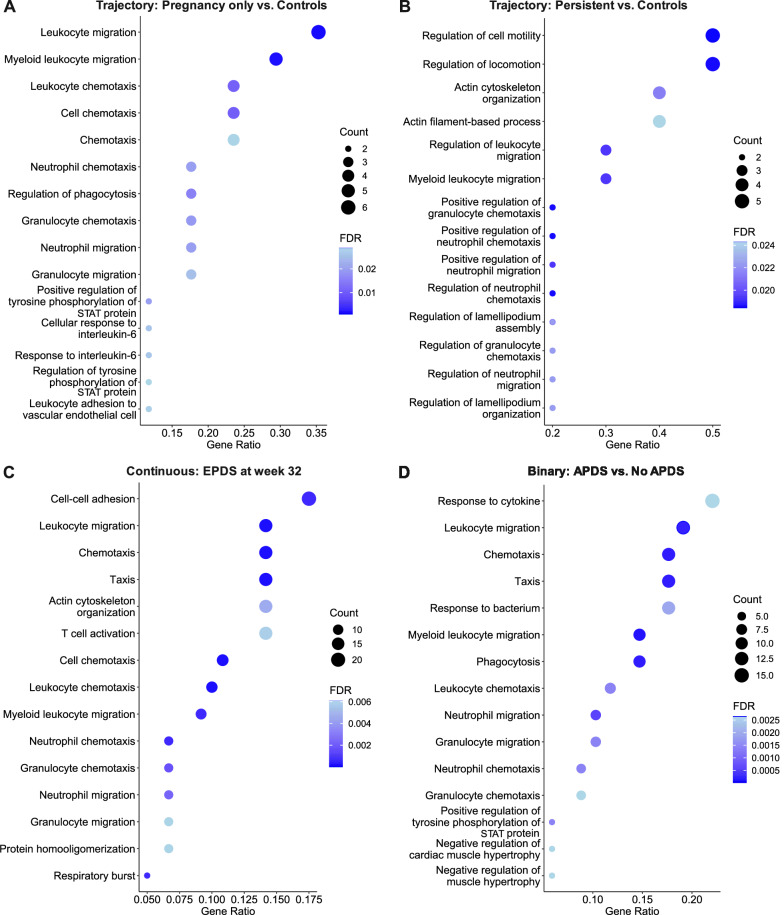
Biological processes in which DEGs in pregnancy samples are involved, as shown by gene set over-representation analysis. Investigating peripartum depression trajectories, **A** and **B** show the biological processes of DEGs associated with depressive symptoms only during pregnancy and persistent depression, respectively. **C** shows biological processes of DEGs for EPDS score at week 32. **D** shows biological processes of DEGs between APDS and no APDS. FDR False Discovery Rate, Gene Ratio: Percentage of significant DEGs in the given biological process.

**Fig. 6:**
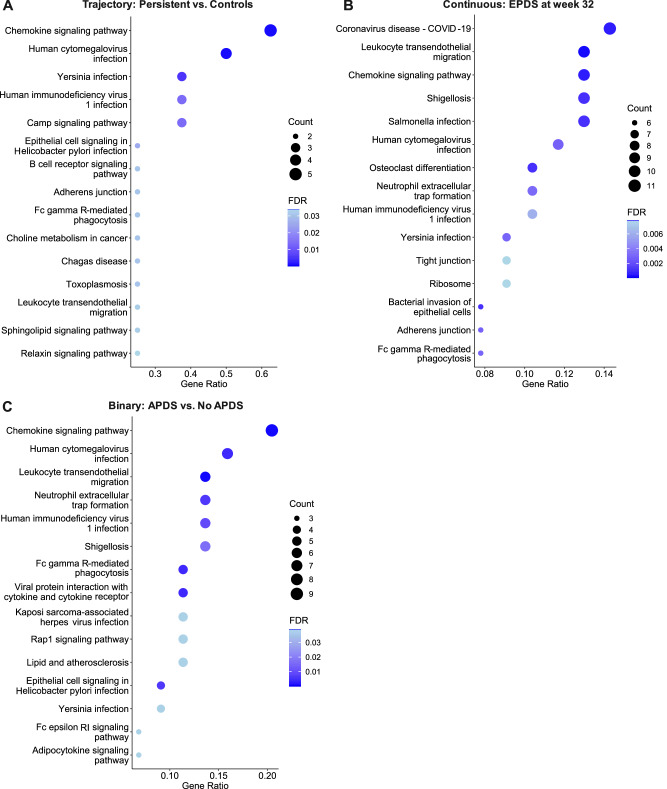
KEGG pathways in which DEGs in pregnancy samples are involved, as shown by gene set over-representation analysis. Investigating peripartum depression trajectories, **A** shows the biological processes of DEGs associated with persistent depression. **B** shows biological processes of DEGs for EPDS score at week 32. **C** shows biological processes of DEGs between APDS and no APDS. FDR False Discovery Rate, Gene Ratio: Percentage of significant DEGs in the given biological process.

Functional annotation of DEGs in depressive symptoms only during pregnancy showed involvement of leukocyte, granulocyte, and neutrophil migration and chemotaxis, positive regulation of tyrosine phosphorylation of STAT protein and cellular response to interleukin-6, and cytokine-mediated signaling pathway (Fig. 5A). Similar pathways were seen when GSEA was performed on genes ranked according to logFC (Supplementary Fig. S2). These DEGs were associated with the KEGG pathway involving interaction of cytokine-cytokine receptor. Regarding persistent depressive symptoms, similar biological processes as for depressive symptoms only during pregnancy were identified (Fig. 5B), and KEGG pathways were involved in chemokine signaling pathway and cAMP signaling pathway (Fig. 6A). When SSRI users were excluded among the depressed groups, top DEGs were similar but did not reach statistical significance; hence, no significant DEGs were seen in the depressive symptoms trajectories vs. controls.

Regarding the individuals with samples collected postpartum, there were no significant DEGs in the group with postpartum-onset depressive symptoms compared to controls, nor among those with persistent depressive symptoms compared to controls.

When SSRI users were excluded among the depressed trajectories, individuals with postpartum-onset depressive symptoms displayed four genes upregulated and one gene downregulated compared to controls (Supplementary Excel File).

### DEGs in relation to EPDS score

Relations between the severity of peripartum depressive symptoms and gene expression were investigated to identify quantitative relationships. In individuals with samples collected during pregnancy, there were 16 upregulated and 109 downregulated DEGs significantly associated with EPDS score at week 32 among samples collected late pregnancy (Figs. 3C and 4C, Supplementary Excel File). Functional analyses showed involvement of cell-cell adhesion, leukocyte migration, chemotaxis, actin cytoskeleton organization and T-cell activation (Fig. 5C). Similar pathways were seen when GSEA was performed on genes ranked according to logFC (Supplementary Fig. S2). Relevant KEGG pathways were leukocyte transendothelial migration, chemokine signaling pathways, and osteoclast differentiation (Fig. 6B). There were no significant DEGs for EPDS score at postpartum week 6.

When SSRI users were excluded from the depressed trajectories, there were eight genes upregulated and 48 genes downregulated associated with EPDS score in week 32. Again, there were no significant DEGs for EPDS score at postpartum week 6.

In individuals with samples collected during postpartum, two upregulated and two downregulated genes associated with EPDS score at postpartum week 6 (Figs. 3E and 4E, Supplementary Excel File). When SSRI users were excluded from the trajectories of peripartum depression, five upregulated and one downregulated gene were associated with EPDS score at postpartum week 6 (Supplementary Excel File). GSEA showed immune-related biological processes (Supplementary Fig. S3).

### DEGs in relation to APDS and PPDS

To facilitate comparison of results with previous studies, transcriptome differences were also investigated in relation to depression as a binary outcome, both in pregnancy and in the postpartum: (1) APDS (depressive symptoms during pregnancy which could be persistent or resolved postpartum) vs. no APDS (controls and postpartum-onset depressive symptoms), and (2) PPDS (depressive symptoms postpartum which could be persistent or postpartum-onset depression) vs. no PPDS (controls and depressive symptoms only during pregnancy) respectively. In individuals with a sample collected during pregnancy, those with APDS had one upregulated and 72 downregulated genes compared to those without APDS (Figs. 3D and 4D, Supplementary Excel File). LogFC of DEGs was in the range of [−0.56, 0.20]. Functional analyses showed involvement in leukocyte and neutrophil migration and chemotaxis, phagocytosis, and negative regulation of cardiac muscle hypertrophy (Fig. 5D). Similarly, immune-related biological processes were found with GSEA (Supplementary Fig. S2). The involved KEGG pathways included the chemokine signaling pathway, the leukocyte transendothelial migration and neutrophil extracellular trap formation, among others (Fig. 6C). There were no significant DEGs in the PPDS vs no PPDS comparison. When SSRI users were excluded from the peripartum depression trajectories, those with APDS had one gene upregulated and 33 genes downregulated compared to those without APDS. Those with PPDS had one gene upregulated compared to those without PPDS.

In individuals with samples collected during postpartum, those with PPDS had six upregulated and two downregulated genes (Figs. 3F and 4F, Supplementary Excel File). When SSRI users were excluded among the peripartum depression trajectories, those with PPDS had six upregulated and one downregulated genes compared to those without PPDS (Supplementary Excel File).

### Replication of previous findings on DEGs associated with PPD

**Fig. 7:**
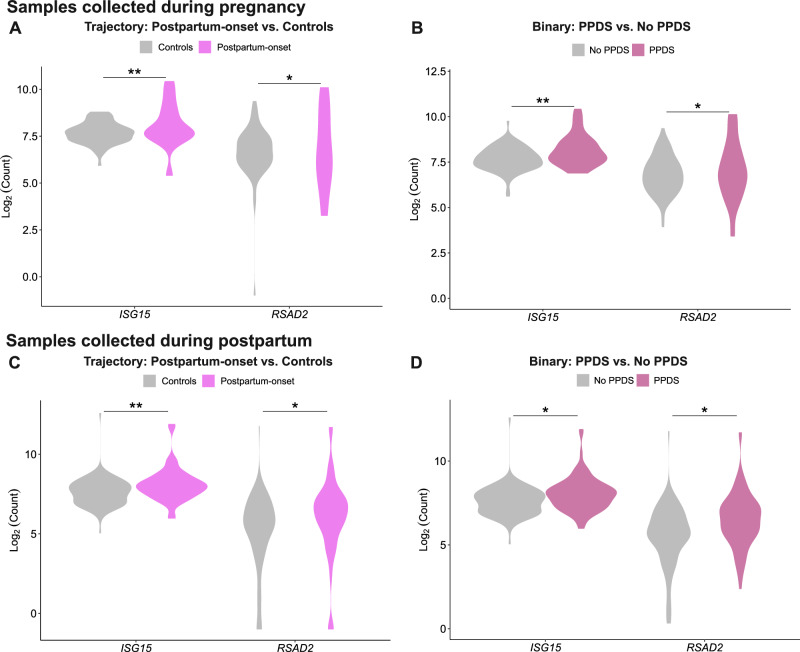
*ISG15* and *RSAD2* expression in postpartum depression. Violin plots showing upregulation of *ISG15* and *RSAD2* in samples collected during pregnancy **A,** **B** and postpartum **C,** **D** when investigating peripartum trajectories **A,** **C** and PPD vs. no PPD **B,** **D**. FDR False Discovery Rate (* FDR < 0.05, ** FDR < 0.01).

We investigated transcripts previously associated with PPD and EPDS scores [20] in an attempt to validate previous results. Moreover, we explored genes expressed in the brain during the peripartum period [25, 26]. Of the 38 PPD, 71 EPDS, and 869 CNS gene transcripts, we identified 11, 19, and 300 gene transcripts in the samples collected during pregnancy. Regarding the PPD genes, 2 out of 11 were significantly upregulated among postpartum-onset depressive symptom cases (*Interferon-stimulated gene 15, ISG15* and *Radical S-Adenosyl Methionine Domain Containing 2, RSAD2*) (Fig. 7A), whereas there were no significant genes associated with depressive symptoms only during pregnancy or with persistent depressive symptoms. None were significantly associated with APDS either, while the same two genes (*ISG15* and *RSAD2*) were significantly upregulated in PPDS (Fig. 7B). For the EPDS genes, none was significantly associated with EPDS score at week 32 or postpartum week 6. Of the CNS genes, none was associated with the peripartum depressive symptoms trajectories, APDS, PPDS, nor EPDS scores at week 32 and postpartum week 6.

Concerning the samples collected postpartum, 11 out of 38 PPD genes, 18 out of 71 EPDS genes, and 366 out of 700 CNS genes were identified in our study. For the PPD genes, the same two genes (*ISG15* and *RSAD2*) were significantly associated with the trajectory postpartum-onset depressive symptoms (Fig. 7C) and with binary PPDS (Fig. 7D). Regardless of the timepoint of sample collection (pregnancy or postpartum), both *ISG15* and *RSAD2* were upregulated in the group with postpartum-onset depression compared to the controls (logFC 0.6–0.8, and 1.1 respectively).

## Discussion

The transcriptomic signatures of depressive symptoms during pregnancy and postpartum were identified. In individuals with samples collected at late pregnancy, symptoms of depression only during pregnancy, as well as persistent depressive sumptoms, were associated with downregulation of 18 and 10 distinct genes, respectively, compared to controls. In the same individuals (sampled at late pregnancy), greater severity of depressive symptoms at gestational week 32, was associated with downregulation of 109 genes, and upregulation of 16 genes. The vast majority of those were related to genes involved in immune-response and cell motility processes. Using this hypothesis-free exploratory approach, no DEGs were found in individuals sampled at late pregnancy that developed depressive symptoms postpartum compared to controls.

Notably, some of the previous findings from [20] were here replicated, even when applying the ≥ 12 cut-off for EPDS compared with ≥ 10 in Mehta et. al. The genes *ISG15* and *RSAD2* involved in chemotaxis and inflammatory processes, cytokine-related pathways, and the innate immune system, were upregulated in postpartum-onset depression cases compared to the controls, in line with the findings by Mehta et al. [20]. Of interest, overexpression of *Isg15* in neonatal rats was shown to impede dendrite development and induced depression-like behaviors [44]. *Rsad2* gene expression was positively correlated with depression-like immobility behaviors in mice [45].

Lastly, no DEGs were found among the different peripartum depressive symptoms trajectories in individuals sampled at postpartum week 8. Nevertheless, in those individuals, increased severity of depressive symptoms at postpartum week 6 was associated with upregulation of two genes and downregulation of two genes.

In line with the present findings of immune-related gene downregulation in depressive symptoms only during pregnancy or in persistent depressive symptoms, downregulation of genes involved in immune or inflammatory pathways has been found in cell lines from females with peripartum depression [21], and in placentas of mothers with high anxiety and depression symptoms during pregnancy [46]. Lower levels of regulatory markers of inflammation in females with APD have also been shown [47]. Taken together, individuals with depression only during pregnancy are likely to have differential transcriptional profiles that may lead to blunted immune-related biological processes during pregnancy compared to unaffected controls [48].

On the other hand, activation of the immune system/higher expression of inflammatory markers has been linked to PPD [14, 49], while both up- and down-regulated genes enriched for immune-responses during late pregnancy have also been associated with PPD [20]. Of relevance, lower levels of extracellular vesicles (EV) mRNA related to autophagy during pregnancy, but higher levels of EV mRNA from ribosomes and mitochondria have been found in postpartum-onset depression cases compared to unaffected controls [23]. The present findings relate thus mostly to immune system pathways, not replicating all earlier findings [50, 51]. Nevertheless, findings in our study are in line with those of Rudzinskas et al. and others, pointing to differences among a broad range of immune-relevant genes differentially regulated in women with PPD/depressive symptoms, an overall down regulation of genes in PPD, and the identification of the DEG, *RSAD2* (Fig. 7), which is involved in immune signalling,  and has been shown to be altered in PPD lymphoblastoid cell lines [21] and postpartum depression [20].

The top five DEGs associated with depressive symptoms only during pregnancy (for samples collected during pregnancy) were *Cytochrome P450 4F3* (*CYP4F3), Leucine-rich α2-glycoprotein 1 (LRG1), Triggering receptor expressed on myeloid cells 1 (TREM1), Sushi domain containing 6 (SUSD6)*, and *Pleckstrin Homology and RhoGEF Domain Containing G3 (PLEKHG3)*. The range of effect was [−0.52, −0.43]. LRG1 is a pro-inflammatory cytokine. Increased circulating LRG1 levels have been associated with neurodegenerative disorders such as dementia, Parkinson’s and Alzheimer disease [52] and chronic social stress exposure in animals [53]. TREM-1 is a glycoprotein of the immunoglobulin superfamily, playing an important role in immune responses [54]. Recently, inhibition of TREM1 alleviated depressive-like behaviors in a mouse model by mitigating neuroinflammation [55]. Herein on the contrary, downregulation of both *LRG1* and *TREM1* was associated with depression only during pregnancy, in line with a possible pregnancy-only related signature. Indeed, findings on depression are often different from those based on the postpartum or outside the perinatal period [56]. One example is the vascular endothelial growth factor A (VEGF-A) which is typically higher in depression outside the perinatal period [57] but has been found to be lower in APD [42], and in PPD compared with persistent depression [49]. Other examples pertain to differences in levels of anti-inflammatory markers [47, 49] as well as corticotropin-releasing hormone (CRH) [11, 58], observed between pregnant and non-pregnant depressed individuals.

The top five DEGs associated with persistent depression (for samples collected during pregnancy) were *Coactosin Like F-Actin Binding Protein 1 (COTL1), C-X-C Motif Chemokine Receptor 2 (CXCR2), Lipopolysaccharide Induced TNF Factor (LITAF), Ubiquitin Conjugating Enzyme E2 R2 (UBE2R2)* and *WASP Actin Nucleation Promoting Factor (WAS)*. The range of effect was [−0.43, −0.29]. Activation of the G-protein coupled receptor CXCR2 by the chemokine CCLX1 has been suggested to induce depression-like symptoms. Recently, inhibition of CXCR2 prevented chronic stress-induced depression-like behaviors in mice, suggesting CXCR2 as a potential novel therapeutic target for patients with depression [59]. Herein on the contrary, downregulation of *CXCR2* mRNA expression was associated with persistent depression. Interestingly, lower *CCLX1 mRNA* expression has been found in the postmortem prefrontal cortex (PFC) of depressed suicidal subjects compared to controls [60].

Only one downregulated gene was identified in both among depressive symptoms only during pregnancy and persistent depression when compared to controls, i.e. *CNN2*, coding for calponin 2. Similarly, *CNN2* was also associated with severity of depressive symptoms at pregnancy week 38, and with APD compared to those without APD (Supplementary Fig. S4). Downregulation of Cnn2 protein in macrophages has been suggested as a mechanism to regulate immune response and as a novel approach for treatment of inflammatory diseases [61]. Our results are in opposite direction compared to previous studies showing the association of upregulated CNN2 with depression and psychosis in human and animal studies [62, 63]. Upregulated serum *CNN2* expression has also been associated with ectopic pregnancies and pre-eclampsia [64, 65].

*PI3* and *TREM1* were also among the top 5 downregulated genes associated with the severity of depressive symptoms at pregnancy week 32, along with *Myosin Light Chain 9 (MYL9), Suppressor Of Cytokine Signaling 3 (SOCS3)* and *Inositol 1,4,5-Trisphosphate Receptor Interacting Protein (ITPRIP)*, with range of effect [−0.32 (MYL9), −0.17 (TREM1)]. Among the top 5 upregulated genes associated with severity of depressive symptoms at pregnancy week 32 were genes belonging in the L Ribosomal Protein (RPL) gene family: *RPL9, RPL39, RPL27*, the *Ribosomal Protein S3A (RPS3A)* and *CD52 Molecule (CD52)*. Downregulation of the *PRL* gene has been associated with major depression in postmortem human brains and chronic stress in mice. Nonetheless, none of the three *RPL* genes identified herein were found altered in the human brain, but in the murine PFC they were downregulated, while *Rps3a2-3* was in fact upregulated [66].

*TREM1* and *CXCR2* were also among the top 5 downregulated genes in APD compared to no APD participants, along with the *Peptidase Inhibitor 3 (PI3), Retinoic Acid Receptor Alpha (RARA)* and *Regulator of G Protein Signaling 2 (RGS2)*, with a range of effect [−0.56 (PI3), −0.35]. *Enah/Vasp-Like (EVL)* was the only upregulated gene in APD participants compared to those with no APD. Upregulation of *EVL* mRNA expression has been associated with breast cancer [67].

Among postpartum samples, upregulation of two genes (*Epithelial-stromal interaction 1* (*EPSTI1*) and *Prolyl 4-hydroxylase beta polypeptide* (*P4HB*)) and downregulation of other two genes [*O-GlcNAcase* (*OGA*) and *oxysterol binding protein like 11* (*OSBPL11*)] were associated with increasing depressive symptoms at postpartum week 8. Genetic variants in proximity to *EPSTI1* have been nominally associated with poorer cognitive therapy outcomes in depressive patients [68], while treatment with the antidepressant venlafaxine has been associated with decreased P4HB mRNA and protein expression during neural differentiation in an in vitro model [69]. Decreased *Oga* mRNA levels have been associated with depression-resistant behaviors in mice [70].

Strengths and limitations should be considered when interpreting the present results. The current study comprises a well-characterized sample using validated psychometric tools and including relevant sociodemographic variables. An important limitation is that no psychiatric interview was performed to investigate lifetime diagnoses. However, good sensitivity and specificity has been demonstrated for the EPDS [12, 29, 30]. This study was nevertheless designed to assess differences between EPDS-positive vs. EPDS-negative individuals and therefore it lacks power to assess differences between controls and those fulfilling criteria for a major depressive episode perinatally. A major strength is the repeated assessment of peripartum depression symptoms throughout pregnancy and postpartum, which allows to study subgroups based on trajectories of symptom onset and persistance. Moreover, having genome-wide gene expression data allowed us to validate findings of previous studies. The analyses based on continuous EPDS scores and the group comparisons based on binary cross-sectional outcomes (i.e. APDS vs. no APDS and PPDS vs. no PPDS) had considerable sample size. On the other hand, the power to identifiy predictive late-pregnancy transcriptomic markers is limited by the small sample size of the group providing a pregnancy blood sample and developing depression symptoms only postpartum (*n* = 14), thus potentially explaining the lack of findings related to this group. Indeed, results should be interpreted with caution and their replication in larger cohorts is highly recommended to investigate the subtle changes in expression associated with depression combined with the variability of transcriptional expression across the population. It should be noted that the high prevalence of cases is dependent on the nested case-control study design; participants with depressive symptoms invited for additional visits, during which RNA samples were collected, were oversampled in comparison to controls.

The use of self-report instruments, as employed herein, can always entail a risk for information bias [71]; even response bias cannot be excluded, when sensitive or potentially embarrassing events are assessed [72]. Assessment of events occurring retrospectively can even posit memory bias [73]. Moreover, standardized self-reported data lack clinical verification and comprehensive patient history, potentially overlooking comorbidities and accurante account of medications’ effects on depressive symptoms. Replication in larger and clinically defined samples is therefore needed.

Further, peripheral measurements (i.e. blood) only partly reflect central measures (mRNA postmortem brain) [74], thus differences found herein cannot be extrapolated to brain differences. Nevertheless, peripheral blood cells are shown to share more than 80% of the transcriptome with the brain [75], while many psychiatric disorders are associated with peripheral inflammation, which seems to affect brain functions and subsequent behavior [23]. Since this study utilized bulk RNAseq of the whole blood, it is important to note that cell-type specific gene expression may be undetected or current results may fluctuate due to the composition of the cell types in the blood [9, 23, 76].

Attrition in the whole of the BASIC study reached 29% at one year postpartum, with higher dropout later than the six months assessed in the current study. Considering that participants in the BASIC cohort exhibited higher educational attainment and lower prevalence of overweight compared to the general population of pregnant women in Sweden [27], the generalizability of these findings should be interpreted with caution.

Exclusion of SSRI users has not been considered by previous studies, but it was applied herein as part of sensitivity analyses. When SSRI-users were excluded from the binary outcome analyses or the analyses with EPDS scores (both of which relied on larger samples), the results remained virtually the same. However, upon exclusion of SSRI users from the depressive symptom trajectories (*n* = 3 for antepartum depression, *n* = 1 for postpartum-onset depression, and *n* = 2 for persistent depression), there were not anymore, any significantly differentiated genes between the trajectories for samples taken during pregnancy. Since the tops DEGs were similar to the significant DEGs in the main analyses, reduced statistical power after the exclusion of those participants from already small-sized groups could explain the above difference. On the other hand, this change might signal that SSRI use might either impact on transcriptome or reflect more severe form of depression. It should be, however, noted that the effect of other types of antidepressants, e.g. SNRI (serotonin and norepinephrine reuptake inhibitors), NaSSA (Noradrenergic and specific serotonergic antidepressants) or TCA (tricyclic antidepressant) was not considered. Finally, only 16 individuals had provided samples both during late pregnancy and postpartum. Future studies assessing the same individual both during pregnancy and postpartum will allow us to identify potential peripartum depression-related intra-individual changes at the transcriptomic level.

## Conclusions

The present findings indicate a link between depressive symptoms during pregnancy only, as well as persisting throughout pregnancy and the postpartum period, and downregulation of the expression of immune-related genes in blood samples collected during late pregnancy. This was further supported by the downregulation of more than a hundred genes, mainly involved in immune-response and cell motility pathways, among those with greater severity of depressive symptoms during late pregnancy. No DEGs of postpartum-onset depressive symptoms were identified in late pregnancy, neither between the different trajectories of peripartum depressive symptoms at two months postpartum in the explorative analyses. Nevertheless, when performing targeted hypothesis-based analyses on previously identified PPD-related DEGs, both at late pregnancy and postpartum, the immune- and inflammation-related genes *ISG15* and *RSAD2* were upregulated in postpartum-onset depressive symptoms cases compared to the controls, in line with [20]. Our results expand on the pathophysiology of APD involving inflammatory dysregulation, confirming the growing body of evidence on the involvement of the immune system in PPD, without pinpointing specific mechanisms. Future studies with larger samples, repeated sampling, and multi-level omics analyses could help pinpoint more specific mechanisms. Possible drug repurposing studies could also be of use in the field. Further, transcriptomic markers alone do not seem to be able to act as biomarkers predicting PPD risk. Future studies might consider combining some top transcriptomic markers with strong background predictors in order to assess if this approach could aid in prediction or early identification of PPD cases.

## Supplementary information

Supplement

## Data Availability

Data are available on Gene Expression Omnibus database repository with the GEO Accession code GSE290313.

## Code availability

The code is available upon reasonable request.
