# Supplementary material for: Differentially expressed transcripts associated with depressive symptoms during pregnancy and postpartum
Source: Mol Psychiatry. 2025 Jun 5;30(10):4736–48. doi: 10.1038/s41380-025-03068-z (PMC12436180; doi:10.1038/s41380-025-03068-z)
Supplement: Supplementary file 1 — Supplement [file 41380_2025_3068_MOESM1_ESM.docx]

***Transcriptomic signatures of peripartum depression trajectories***

Richelle D. Björvang, Maria Vrettou, Xabier Bujanda Cundin, Eugenio Del Prete, Joëlle Rüegg, Susanne Lager, Diego di Bernardo, Erika Comasco, Alkistis Skalkidou

**Supplementary material**

**RNA analyses**

RNA was extracted using the PAXgene 96 Blood RNA Kit (Qiagen GmbH) according to manufacturer’s instructions. Sample concentrations were determined using spectrophotometry (Nanodrop and Dropsense, Unchained Labs) and RNA integrity was analyzed using capillary electrophoresis (Fragment Analyzer), considering RNA Integrity Number greater than 7. Total RNA was prepared for sequencing using QuantSeq 3’ mRNA-Seq Library Prep Kit FWD (Lexogen) according to manufacturer’s instructions. The RNA libraries were sequenced as 1 x 100 bp reads on Illumina NovaSeq 6000 S2 Flow CellSystem. Raw RNA-Seq reads were preprocessed by removing adapters and low-quality sequences using BBDuk function from *BBTools* version 39.01 (27) with the following parameters: k=13, ktrim=r, useshortkmers=t, mink=5, qtrim=r, trimq=10, minlength=20. Then sequences were aligned to the UCSC Genome Browser hg38 using *Spliced Transcripts Alignment to a Reference* (STAR) with standard parameters. Finally, the count matrix per gene was obtained for each sample using *htseq-count* function from *HTSeq* (28) with the following parameters: -m intersection-nonempty -s "yes".

For the samples collected during pregnancy, 3,745 out of 19,259 protein-coding genes in the reference set (37) were not expressed in any sample, while 7,626 protein-coding genes were detected in at least 12 samples with a minimum of 50 reads. For the samples collected postpartum, 3181 out of 19,259 protein-coding genes were not expressed in any sample, while 9,060 protein-coding genes were detected in at least 12 samples with a minimum of 50 reads

Figures

Figure S1. Flowchart of study overview

Figure S2. Biological Processes for genes arranged by log-fold change (pregnancy w38) associated with (A) depression only during pregnancy, (B) postpartum-onset depression, (C) persistent depression, (D) APD vs no APD, (E) PPD vs. no PPD, (F) EPDS score at pregnancy week 32, (G) EPDS score at postpartum week 8.

Figure S3. Biological Processes for genes arranged by log-fold change (postpartum w8) associated with (A) persistent depression, (B) EPDS score at postpartum week 8, (C) PPD vs. no PPD. Biological Processes whose enrichment score was above the absolute value of 0.75 were plotted.

Figure S4. Venn diagram of DEGs in pregnancy samples.

Tables

Table S1. Key findings of studies investigating peripheral mRNA levels in relation to perinatal depression

Table S2. Characteristics of participants with pregnancy samples

Table S3. Characteristics of participants with postpartum samples

BASIC

(6541 pregnancies

in 5480 women)

Invitations sent for additional visit at week 38 pregnancy (n=715) and postpartum week 8 (n=713), resulting to 349 (49% participation rate) pregnancy and 413 (58% participation rate) postpartum sessions

Exclude: Twin pregnancies (n=2),

missing information on EPDS trajectory (n=1), smoking during sample collection (n=1),

no pregnancy RNA sample (n=221),

no postpartum RNA sample (n=199)

Women with RNA sample during pregnancy (n=110)

Women with RNA sample during postpartum (n=195)

Women with RNA sample both during pregnancy and postpartum (n=18)

Exclude: RIN<7 (n=21 samples),

read count<1M (n=2 samples),

outliers based on PCA plot (n=7 samples),

controls using SSRI when sample was taken (n=6 samples)

Women with RNA sample during pregnancy (n=101)

Women with RNA sample during postpartum(n=172)

Women with RNA sample both during pregnancy and postpartum (n=16)

Figure S1. Flowchart of study overview

*
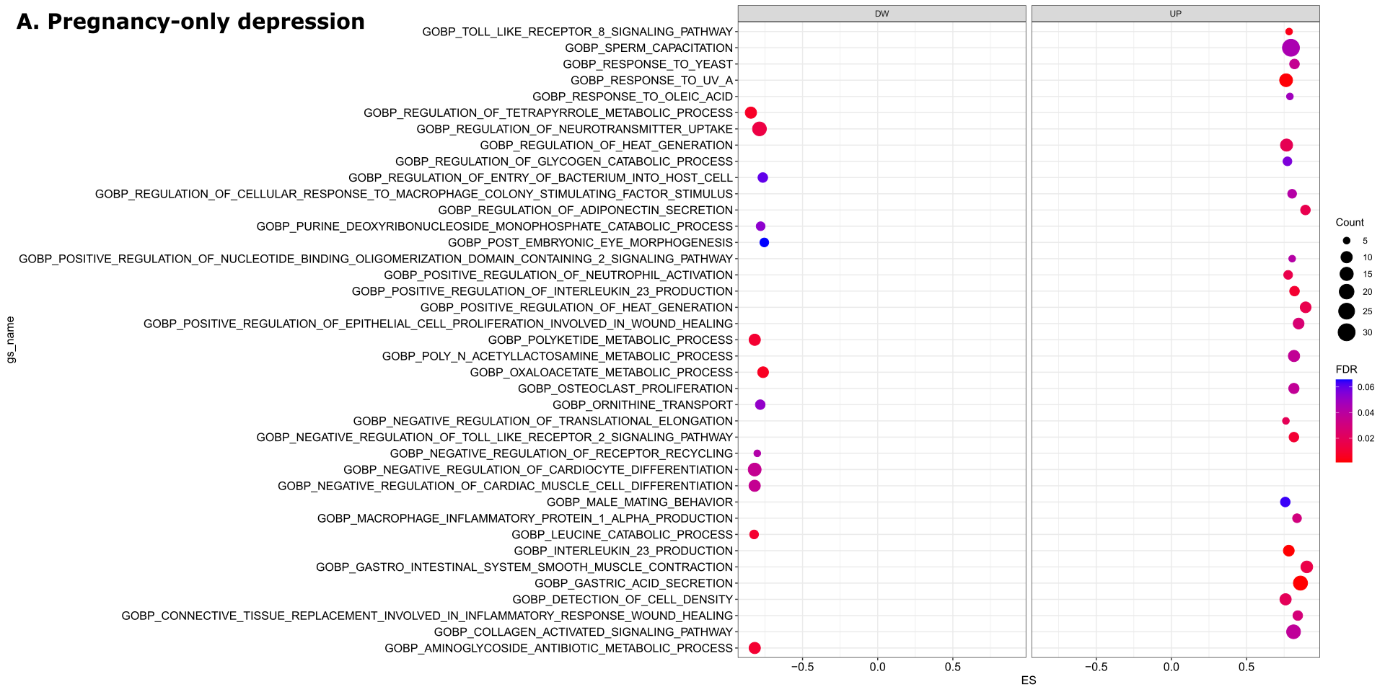
*

*
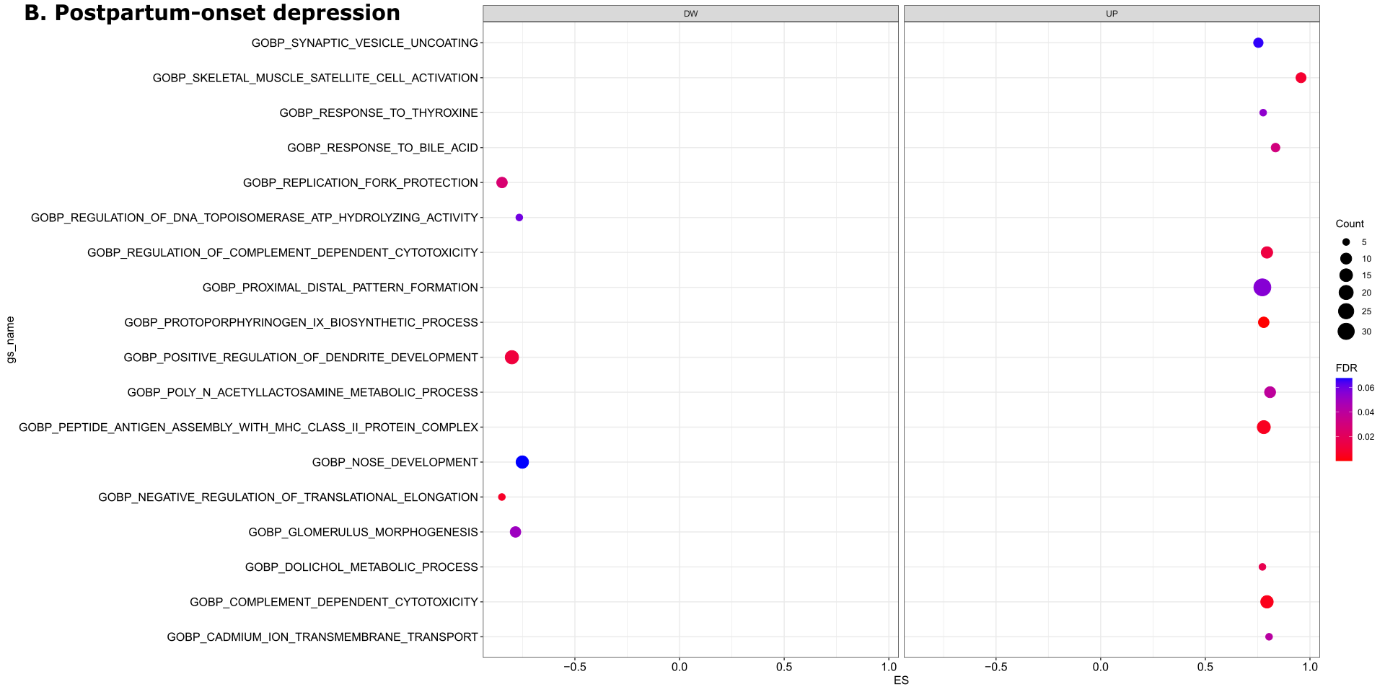
*

*
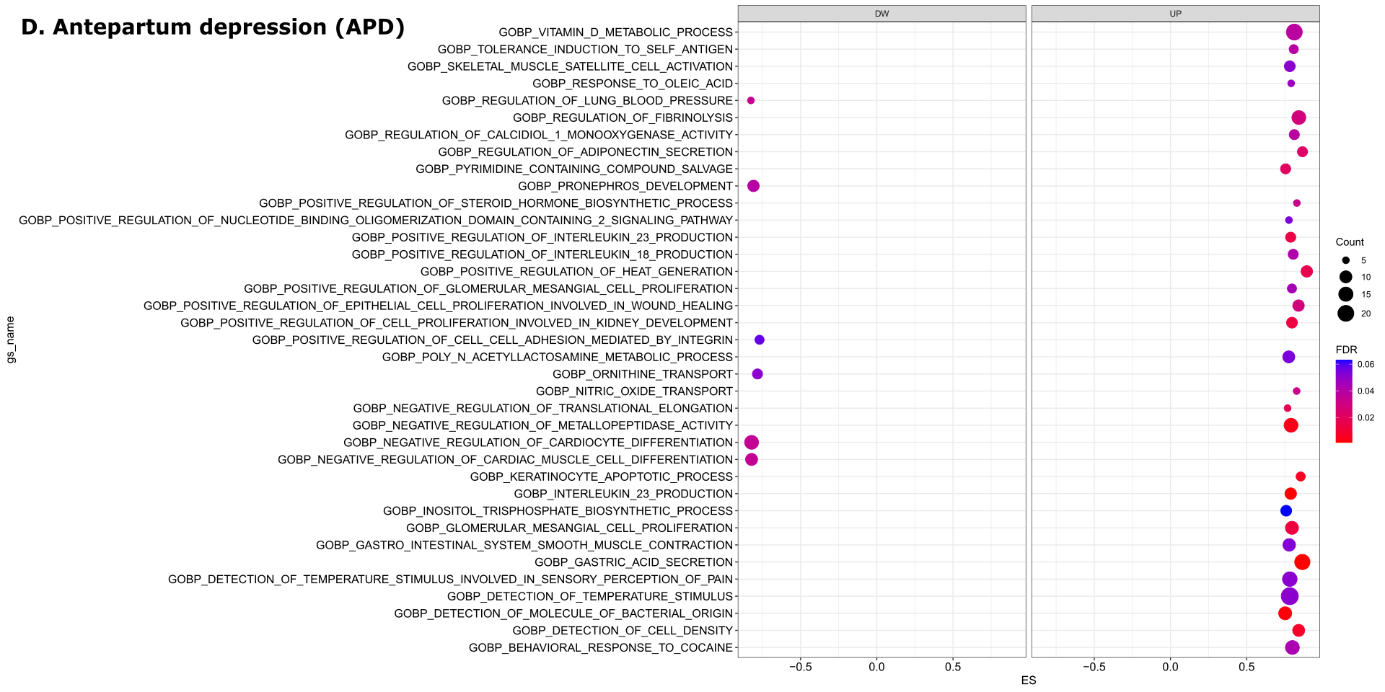
*

*
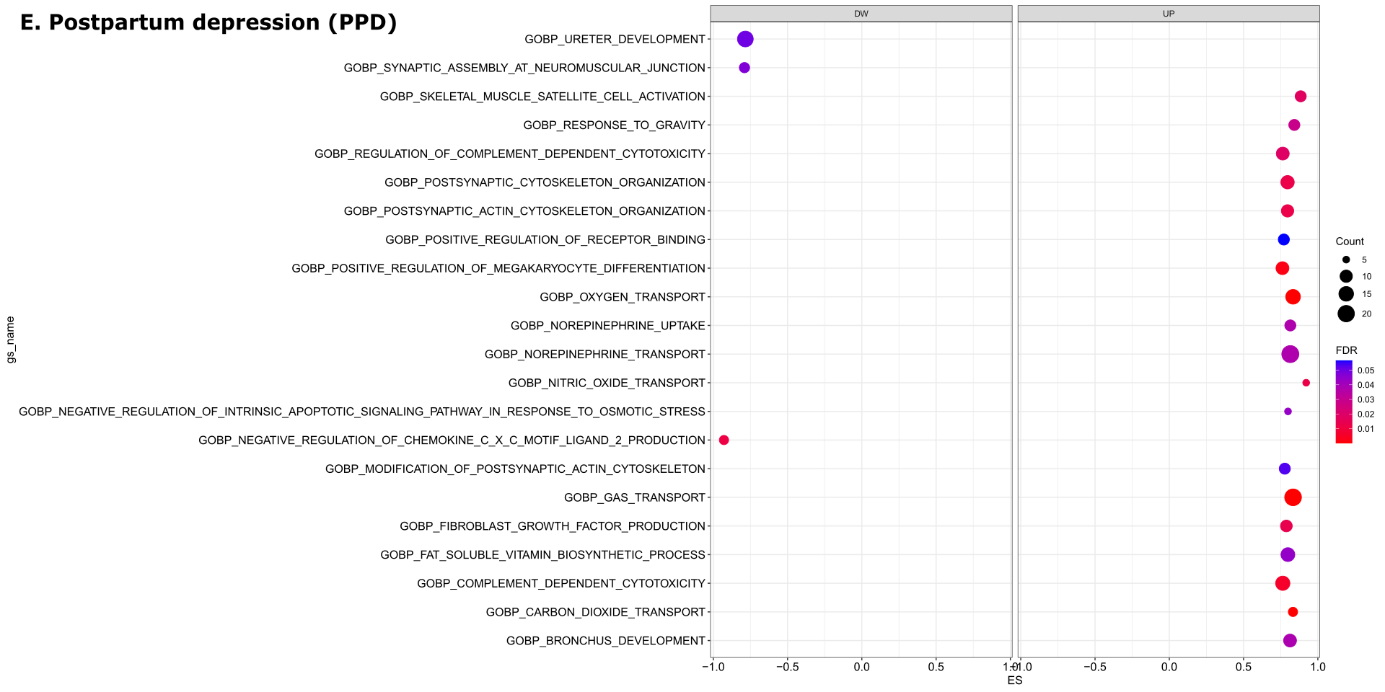
*

*
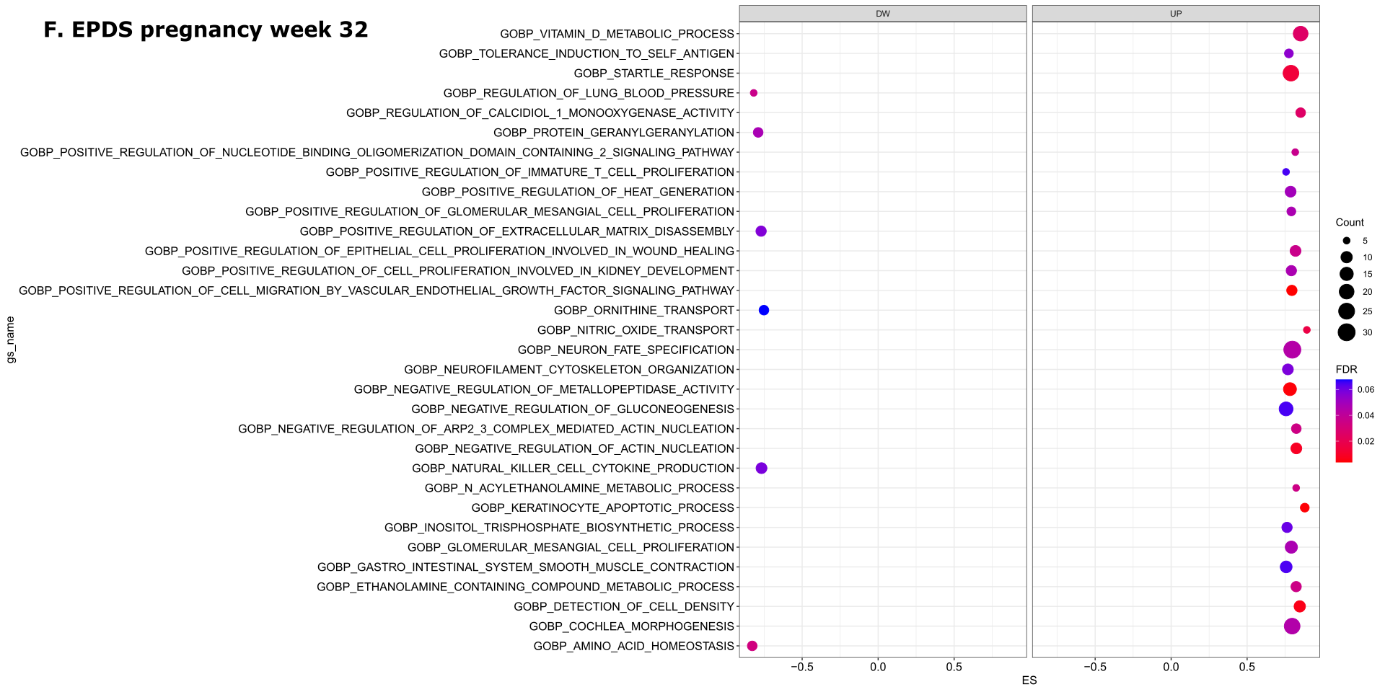
*

*
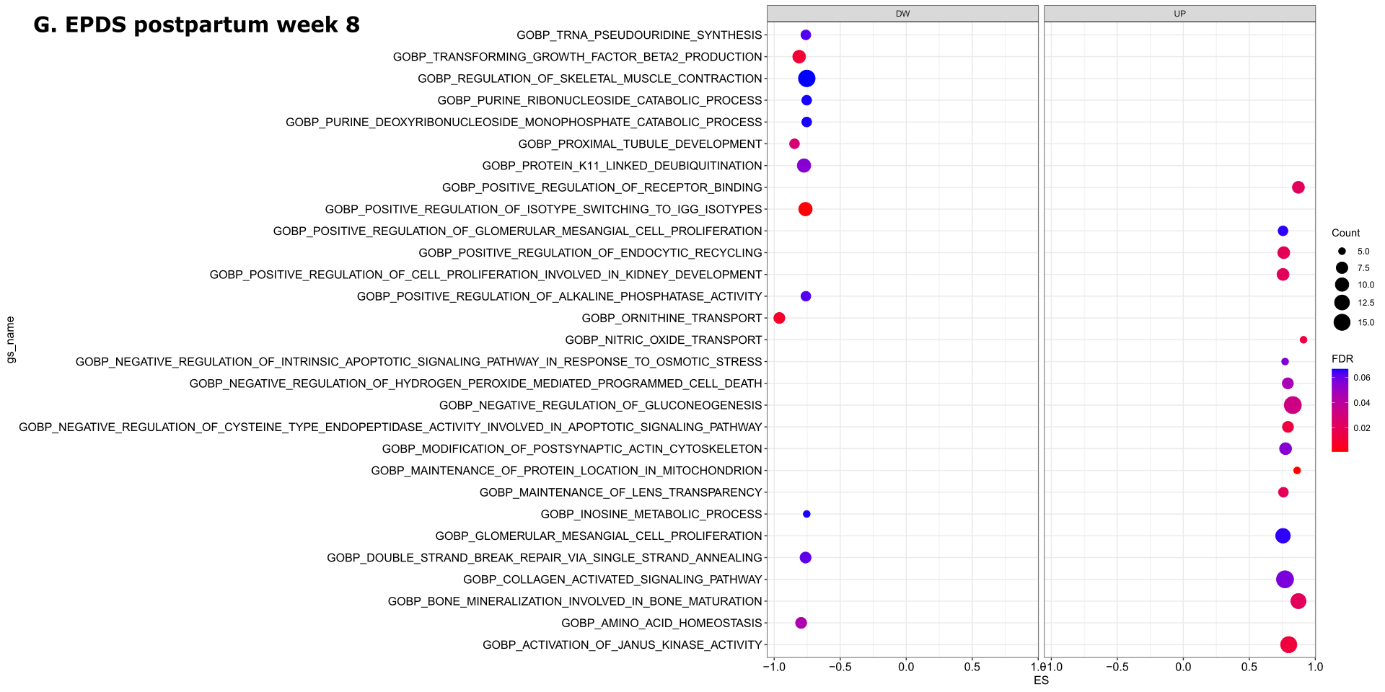
*

**Figure S2. Biological Processes for genes arranged by log-fold change (pregnancy w38) associated with (**A) depression only during pregnancy, (B) postpartum-onset depression, (C) persistent depression, (D) APD vs no APD, (E) PPD vs. no PPD, (F) EPDS score at pregnancy week 32, (G) EPDS score at postpartum week 8. Biological Processes whose enrichment score was above the absolute value of 0.75 were plotted.

**
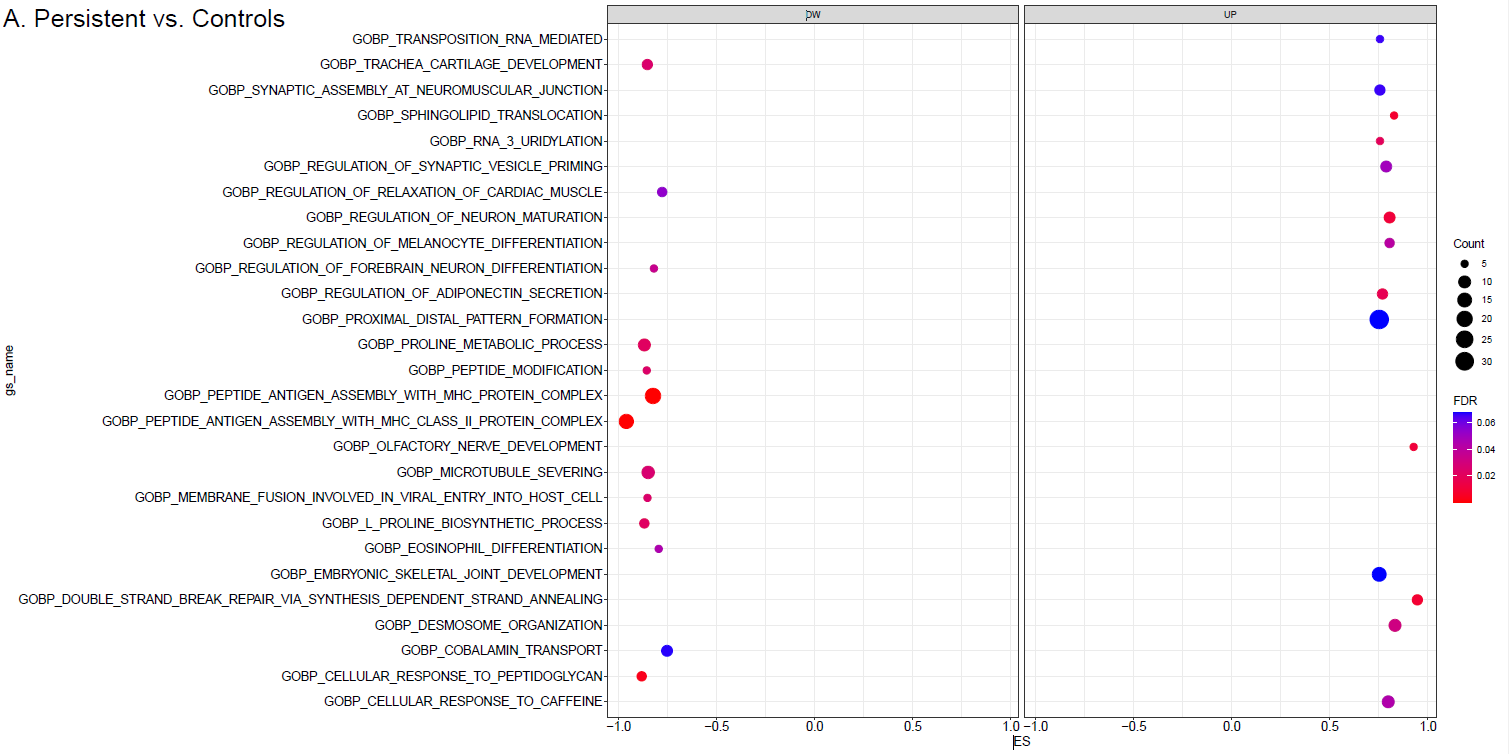
**

**
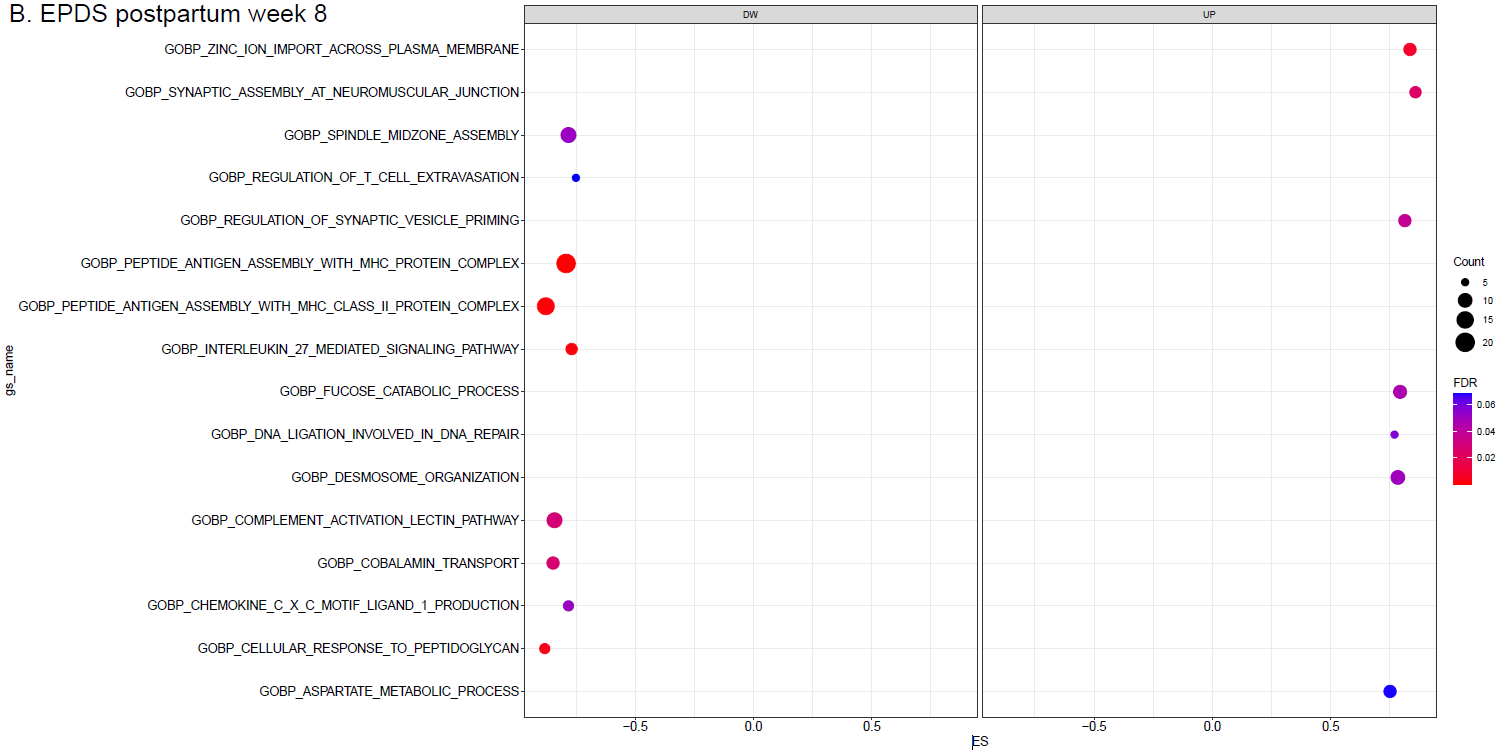
** **
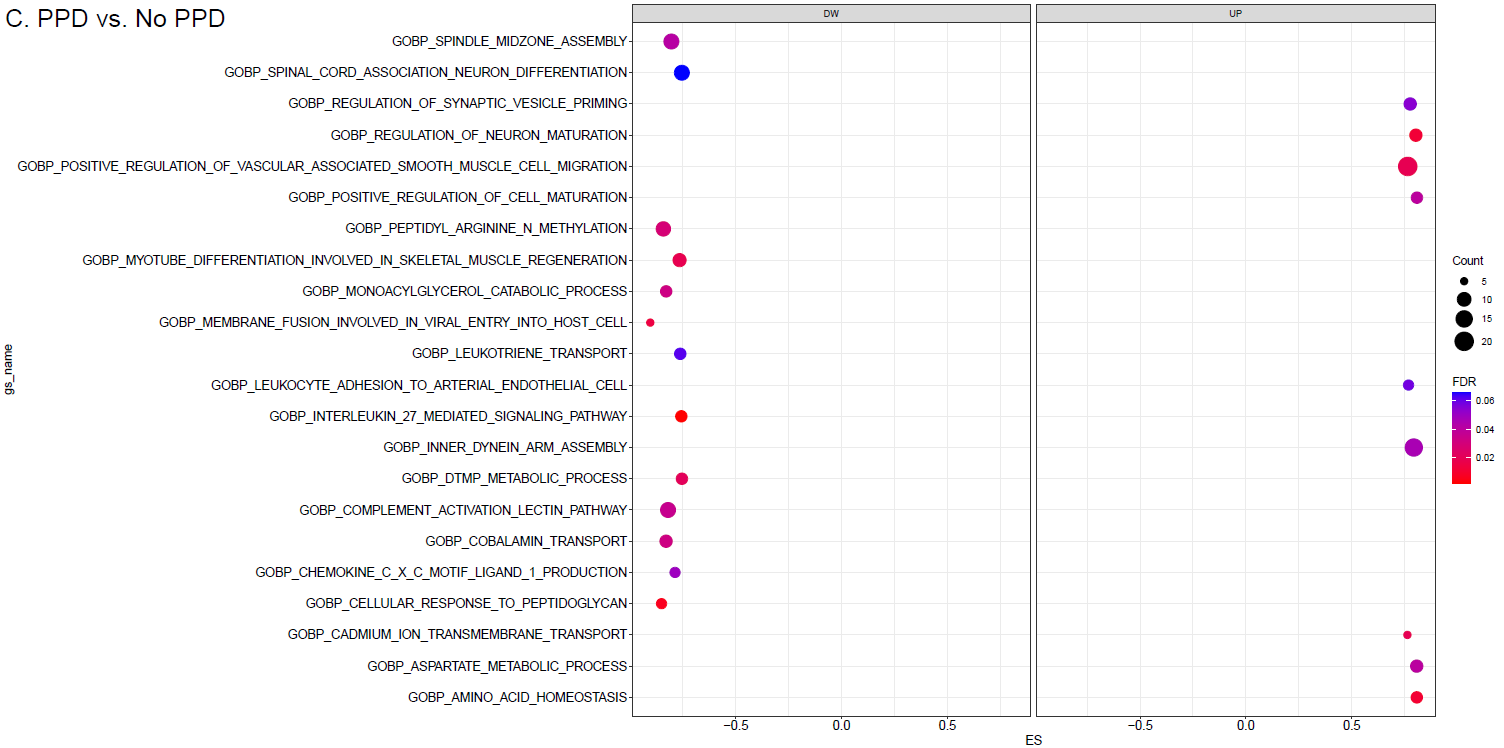
**

**Figure S3. Biological Processes for genes arranged by log-fold change (postpartum w8) associated with** (A) persistent depression, (B) EPDS score at postpartum week 8, (C) PPD vs. no PPD. Biological Processes whose enrichment score was above the absolute value of 0.75 were plotted.

**
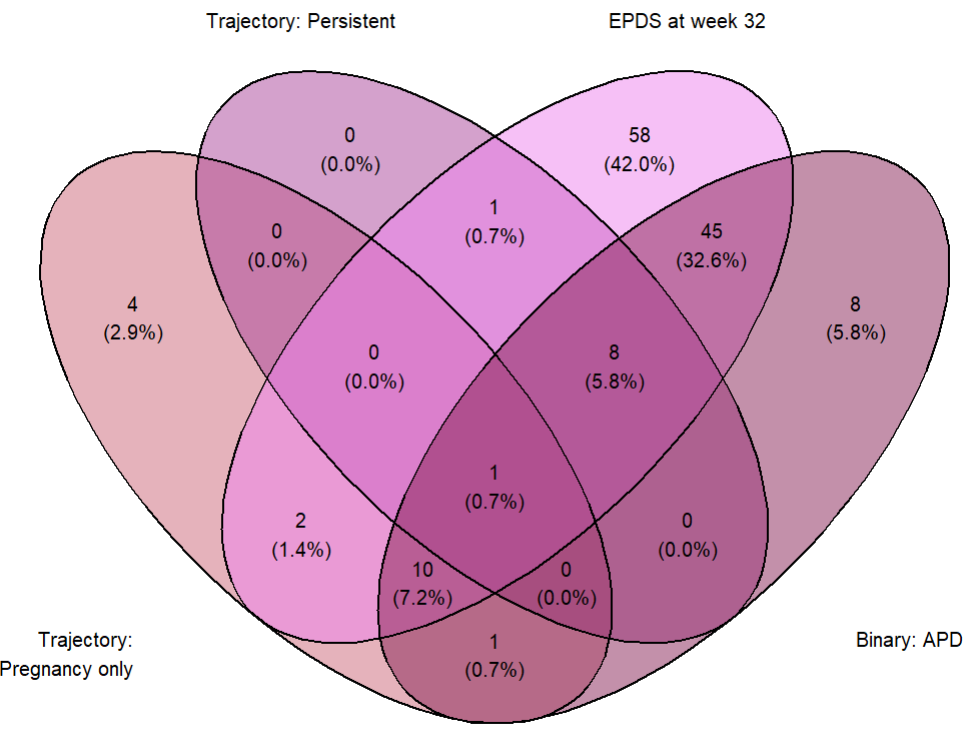
**

**Figure S4. Venn diagram of DEGs in pregnancy samples.** Number of similar and different DEGs in pregnancy only vs. controls, persistent vs. controls, EPDS score at week 32, and APD vs. No APD. *CNN2* is the gene common to all groups.

**Table S1. Key findings of studies investigating peripheral mRNA levels in relation to perinatal depression**

Table

| **Study** | **N** | **Time points** | **Postpartum depression** | **Tissue; mRNA analysis** | **Findings** |
| --- | --- | --- | --- | --- | --- |
| Smirnova et  al., 2024 | 14 (7 cases, 7 controls) | gt 2 and 3;  ppw 2 and 6; ppm 3 and 6 | EPDS (cut-off ≥ 13) | Plasma EVs mRNA; RNA-sequencing | Postpartum depression (PPD) was associated with the EV mRNA levels of 13 female brain-specific transcripts |
| Osborne et  al., 2022 | 14 (7 euthymic in pregnancy with postpartum onset cases, 7 controls) + 28 replication sample [14 (9 euthymic + 5 depressed in pregnancy) cases, 14 controls) | gt2 and 3;  ppw 2 and 6; ppm 3 and 6 | EPDS (cut-off ≥ 13) | Plasma EVs mRNA; RNA-sequencing | EV mRNA communication is altered during pregnancy and the postpartum period in euthymic women who go on to develop postpartum depression (PPD). Reduced autophagy was associated with PPD cases. |
| Mehta et al.,  2021 | 137 (15 cases, 122 controls) | gt 3, ppm2 | EPDS (cut-off ≥ 10) | Whole blood; RNA sequencing (gt3) | Postpartum depression scores at 2 months were significantly associated with 71 genes, most of them involved in immune response-related biological processes. Genes previously associated with estrogen sensitivity in PPD were also validated. |
| Mehta et al.,  2014 | 62 (+24 replication cohort) | gt 1 and 3, ppw 7 | BDI  EPDS  HAMD-17 | Whole blood; Microarrays  (gt 1 and 3, ppw 7) | Differentially expressed transcripts (N=116) between the PPD and euthymic women at gt3, enriched for estrogen signaling. The transcripts allowed for 88% prediction of PPD at ppw7. |
| Guintivano et al.,  2022 | 1341 (482 cases, 859 controls) | Ppw6 | MINI | Whole blood; RNA sequencing | Altered transcripts (N=891, representing 789 genes) in B-cells, implicated in B-cell activation and insulin resistance |
| Rudzinskas et al.,  2023 | 20 (9 with past PPD, 11 controls) | ppm 12 | IDAS | Lymphoblastoid cell lines in hormone conditions mimicking pregnancy and parturition; RNA sequencing | Vastly downregulated genes in PPD cases, especially upon supraphysiological estradiol & progesterone addback condition; genes involved in cellular stress regulation were altered |
| Katz et al., 2012 | 106 (128 mRNA samples: 49 cases, 79 controls) | Preconception, gt 1-3 | BDI | Whole blood; real-time PCR | Depressive symptoms diminished the pregnancy-related upregulation in mRNA expression of glucocorticoid-receptor co-chaperone genes |
| Pan et al., 2018 | 83 (56 cases, 27 controls) | Ppw6 | EPDS (cut-off ≥ 10) | PBMCs; RNA sequencing | PPD was positively correlated with genes involved in energy metabolism, neurodegenerative diseases and immune response, and negatively correlated with genes in mismatch repair and cancer-related pathways |

BDI: Beck Depression Inventory; EPDS: Edinburgh Postnatal Depression Scale; EV: extracellular vesicles; HAMD-17: Hamilton Depression Rating Scale-17; IDAS: Inventory of Depression and Anxiety Symptoms; Gt: gestational trimester; gw: gestational week; MINI: Mini International Psychiatric Interview; PBMCs: peripheral blood mononuclear cells; PCR: Polymerase Chain Reaction; ppw; postpartum week; ppm: postpartum month; ppy: post-partum year

**Table S2 Characteristics of participants with pregnancy samples**

|  |  | **Total (N=117)** | **Controls (N=60)** | **Depression only during pregnancy  (N=18)** | **Postpartum-onset depression (N=14)** | **Persistent depression (N=25)** | **P-value** | |
| --- | --- | --- | --- | --- | --- | --- | --- | --- |
| **BACKGROUND** |  |  |  |  |  |  |  | |
| **Age,** median (min, max) |  | 32.0 [21.0, 42.0] | 32.0 [25.0, 41.0] | 30.0 [24.0, 42.0] | 32.0 [21.0, 40.0] | 32.0 [25.0, 38.0] | 0.80 | |
| **Pre-pregnancy BMI,** median (min, max) |  | 23.1 [18.0, 39.8] | 23.1 [18.0, 39.8] | 24.1 [19.8, 29.4] | 23.2 [18.8, 37.4] | 22.9 [18.2, 39.2] | 0.60 | |
| **Nulliparous,** n (%) |  | 58 (49.2%) | 32 (53.3%) | 7 (38.9%) | 4 (28.6%) | 14 (56.0%) | 0.40 | |
| **University education,** n (%) |  | 87 (73.7%) | 43 (71.7%) | 12 (66.7%) | 10 (71.4%) | 21 (84.0%) | 0.78 | |
| **Working/Studying**, n (%) |  | 104 (88.1%) | 53 (88.3%) | 15 (83.3%) | 13 (92.9%) | 22 (88.0%) | 0.52 | |
| **Married/Cohabiting,** n (%) |  | 108 (91.5%) | 55 (91.7%) | 16 (88.9%) | 14 (100%) | 22 (88.0%) | 0.80 | |
| **Smoking before pregnancy,** n (%) |  | 41 (34.7%) | 16 (26.7%) | 9 (50.0%) | 4 (28.6%) | 11 (44.0%) | 0.28 | |
|  |  |  |  |  |  |  |  | |
| **MENTAL HEALTH** |  |  |  |  |  |  |  | |
| **History of depression**, n (%) |  | 48 (40.7%) | 17 (28.3%) | 10 (55.6%) | 6 (42.9%) | 15 (60.0%) | **0.03** | |
| **Anxiety during pregnancy,** n (%) |  | 67 (56.8%) | 18 (30.0%) | 17 (94.4%) | 7 (50.0%) | 24 (96.0%) | **<0.001** | |
| **SSRI use during pregnancy**, n(%) |  | 6 (5.1%) | 0 (0%) | 3 (16.7%) | 1 (7.1%) | 2 (8.0%) | **0.01** | |
| **Anxiety postpartum,** n (%) |  | 59 (50.0%) | 16 (26.2%) | 10 (55.6%) | 11 (78.6%) | 22 (88.0%) | **<0.001** | |
| **SSRI use postpartum,** n (%) |  | 10 (8.5%) | 1 (1.6%) | 3 (16.7%) | 1 (7.1%) | 5 (20.0%) | **0.03** | |
| **Lifetime trauma events,** median (min-max) |  | 3.00 [0, 11.0] | 3.00 [0, 11.0] | 3.00 [0, 10.0] | 5.00 [1.00, 10.0] | 3.50 [0, 11.0] | 0.24 | |
| **Sense of coherence score,** median (min-max) |  | 144 [75.0, 188] | 159 [130, 188] | 132 [96.0, 180] | 129 [95.0, 170] | 114 [75.0, 149] | **<0.001** | |
|  |  |  |  |  |  |  |  | |
| **PERIPARTUM-RELATED** | | | | | | | |  |
| **PMS/PMDD,** n (%) |  | 11 (9.3%) | 4 (6.7%) | 0 (0%) | 2 (14.3%) | 5 (20.0%) | 0.10 | |
| **Planned pregnancy**, n (%) |  | 97 (82.2%) | 50 (83.3%) | 17 (94.4%) | 8 (57.1%) | 22 (88.0%) | 0.08 | |
| **Sleep >8 hours,** n (%) |  | 41 (34.7%) | 22 (36.7%) | 6 (33.3%) | 4 (28.6%) | 9 (36.0%) | 0.97 | |
| **Fear of childbirthchildbirth,** n (%) |  | 37 (31.4%) | 10 (16.7%) | 7 (38.9%) | 4 (28.6%) | 15 (60.0%) | **0.003** | |
| **Pregnancy complications,** n (%) |  | 65 (55.1%) | 29 (48.3%) | 12 (66.7%) | 5 (35.7%) | 18 (72.0%) | 0.26 | |
| **Positive delivery experience,** n(%) |  | 96 (81.4%) | 47 (78.3%) | 16 (88.9%) | 11 (78.6%) | 21 (84.0%) | 0.29 | |
| **Mode of delivery,** n(%) |  |  |  |  |  |  |  | |
| Vaginal delivery |  | 90 (76.3%) | 42 (70%) | 16 (88.9%) | 12 (85.7%) | 19 (76.0%) | 0.71 | |
| Vacuum extraction |  | 10 (8.5%) | 6 (10%) | 0 (0%) | 1 (7.1%) | 3 (12.0%) |  | |
| Planned CS |  | 9 (7.6%) | 5 (8.3%) | 2 (11.1%) | 1 (7.1%) | 1 (4.0%) |  | |
| Emergency CS |  | 9 (7.6%) | 7 (11.7%) | 0 (0%) | 0 (0%) | 2 (8.0%) |  | |
| **Premature birth,** n (%) |  | 0 (0%) | 0 (0%) | 0 (0%) | 0 (0%) | 0 (0%) | - | |
| **Admission to NICU,** n (%) |  | 7 (5.9%) | 4 (6.7%) | 1 (5.6%) | 1 (7.1%) | 1 (4.0%) | 0.93 | |
| **Breastfeeding,** n (%) |  | 107 (90.7%) | 54 (90.0%) | 17 (94.4%) | 13 (92.9%) | 22 (88.0%) | 0.78 | |

Among the individuals with samples collected during pregnancy, those with depressive symptoms more often reported a history of depression, perinatal anxiety and more early life traumatic events, as well as had a higher percentage of fear of childbirth compared to controls.

**Table S3. Characteristics of participants with postpartum samples**

|  |  | **Total (N=188)** | **Controls (N=98)** | **Depression only during pregnancy**  **(N=4)** | **Postpartum-onset depression (N=48)** | **Persistent depression (N=38)** | **P-value** |
| --- | --- | --- | --- | --- | --- | --- | --- |
| **BACKGROUND** |  |  |  |  |  |  |  |
| **Age,** median (min, max) |  | 31.0 [19.0, 42.0] | 31.0 [22.0, 42.0] | 31.0 [29.0, 39.0] | 30.5 [21.0, 40.0] | 30.0 [19.0, 41.0] | 0.44 |
| **Pre-pregnancy BMI,** median (min, max) |  | 23.4 [17.5, 42.2] | 22.8 [17.9, 42.2] | 24.9 [22.0, 26.2] | 23.8 [17.5, 37.4] | 24.8 [18.7, 39.2] | 0.11 |
| **Nulliparous,** n (%) |  | 87 (46.5%) | 48 (49%) | 2 (50.0%) | 25 (52.1%) | 13 (34.2%) | 0.48 |
| **University education,** n (%) |  | 137 (73.3%) | 71 (72.4%) | 3 (75.0%) | 39 (81.3%) | 25 (65.8%) | 0.25 |
| **Working/Studying**, n (%) |  | 162 (86.6%) | 87 (88.8%) | 3 (75.0%) | 44 (91.7%) | 29 (76.3%) | **0.04** |
| **Married/Cohabiting,** n (%) |  | 183 (97.9%) | 97 (99.0%) | 4 (100%) | 47 (97.9%) | 36 (94.7%) | 0.12 |
| **Smoking before pregnancy,** n (%) |  | 59 (31.6%) | 22 (22.4%) | 2 (50.0%) | 18 (37.5%) | 17 (44.7%) | **0.02** |
|  |  |  |  |  |  |  |  |
| **MENTAL HEALTH** |  |  |  |  |  |  |  |
| **History of depression**, n (%) |  | 71 (38.0%) | 16 (16.3%) | 2 (50.0%) | 25 (52.1%) | 28 (73.7%) | **<0.001** |
| **Anxiety during pregnancy,** n (%) |  | 87 (46.5%) | 23 (23.5%) | 3 (75.0%) | 27 (56.3%) | 35 (92.1%) | **<0.001** |
| **SSRI use during pregnancy**, n(%) |  | 7 (3.7%) | 1 (1%) | 0 (0%) | 2 (4.2%) | 5 (13.2%) | **0.002** |
| **Anxiety postpartum,** n (%) |  | 103 (55.1%) | 26 (26.8%) | 3 (75.0%) | 39 (81.3%) | 35 (92.1%) | **<0.001** |
| **SSRI use postpartum,** n (%) |  | 22 (11.8%) | 0 (0%) | 1 (25.0%) | 8 (16.7%) | 13 (34.2%) | **<0.001** |
| **Lifetime trauma events,** median (min-max) |  | 3.00 [0, 13.0] | 3.00 [0, 11.0] | 7.00 [2.00, 8.00] | 4.00 [0, 11.0] | 5.00 [0, 13.0] | **0.005** |
| **Sense of coherence score,** median (min-max) |  | 150 [62.0, 186] | 160 [126, 186] | 131 [103, 170] | 144 [108, 181] | 118 [62.0, 167] | **<0.001** |
| **PERIPARTUM-RELATED** |  |  |  |  |  |  |  |
| **PMS/PMDD,** n (%) |  | 15 (8.0%) | 3 (3.1%) | 1 (25.0%) | 5 (10.4%) | 6 (15.8%) | **0.02** |
| **Planned pregnancy**, n (%) |  | 138 (73.8%) | 83 (84.7%) | 3 (75.0%) | 32 (66.7%) | 21 (55.3%) | **<0.001** |
| **Sleep >8 hours,** n (%) |  | 56 (29.9%) | 34 (34.7%) | 0 (0%) | 9 (18.8%) | 13 (34.2%) | 0.13 |
| **Fear of childbirthchildbirth,** n (%) |  | 51 (27.3%) | 18 (18.4%) | 2 (50.0%) | 15 (31.3%) | 16 (42.1%) | **0.02** |
| **Pregnancy complications,** n (%) |  | 105 (56.1%) | 46 (46.9%) | 4 (100%) | 29 (60.4%) | 26 (68.4%) | **0.02** |
| **Positive delivery experience,** n(%) |  | 155 (82.9%) | 87 (88.8%) | 4 (100%) | 37 (77.1%) | 28 (73.7%) | **0.01** |
| **Mode of delivery,** n(%) |  |  |  |  |  |  |  |
| Vaginal delivery |  | 140 (74.9%) | 79 (80.6%) | 3 (75.0%) | 32 (66.7%) | 27 (71.1%) | 0.38 |
| Vacuum extraction |  | 10 (5.3%) | 4 (4.1%) | 0 (0%) | 3 (6.3%) | 3 (7.9%) |  |
| Planned CS |  | 12 (6.4%) | 7 (7.1%) | 0 (0%) | 2 (4.2%) | 3 (7.9%) |  |
| Emergency CS |  | 25 (13.4%) | 8 (8.2%) | 1 (25.0%) | 11 (22.9%) | 5 (13.2%) |  |
| **Premature birth,** n (%) |  | 8 (4.3%) | 1 (1.0%) | 1 (25.0%) | 3 (6.3%) | 3 (7.9%) | **0.03** |
| **Admission to NICU,** n (%) |  | 29 (15.5%) | 14 (14.3%) | 1 (25.0%) | 8 (16.7%) | 6 (15.8%) | 0.87 |
| **Breastfeeding,** n (%) |  | 175 (93.6%) | 93 (94.9%) | 4 (100%) | 44 (91.7%) | 35 (92.1%) | 0.61 |

Among the individuals with samples collected postpartum, those with depressive symptoms postpartum compared to those who never had depressive symptoms perinatally were more often unemployed or on parental/sick leave, smokers before pregnancy, had a history of depression, reported perinatal anxiety and more early life traumatic events, and had higher rates of PMS/PMDD, unplanned pregnancies, fear of childbirth, pregnancy complications, negative delivery experience, and premature birth.
